# Comprehensive identification of key compounds in different quality grades of soy sauce-aroma type baijiu by HS-SPME-GC-MS coupled with electronic nose

**DOI:** 10.3389/fnut.2023.1132527

**Published:** 2023-03-07

**Authors:** Junhai Wu, Renyuan Chen, Xiaobo Li, Zheyang Fu, Chun Xian, Wenwu Zhao, Cheng Zhao, Xinying Wu

**Affiliations:** ^1^School of Liquor and Food Engineering, Guizhou University, Guiyang, China; ^2^Key Laboratory of Fermentation Engineering and Biological Pharmacy of Guizhou Province, Guiyang, China; ^3^Guizhou Academy of Liquor Quality Inspection and Testing, Renhuai, China

**Keywords:** soy sauce-aroma type baijiu, different quality grades of base liquor, differential compounds, HS-SPME-GC-MS, E-nose, correlation analysis

## Abstract

In the production of soy sauce-aroma type baijiu (SSAB), the quality of base liquor significantly affects the finished liquor’s quality. Moreover, low-quality liquor may cause health problems. The different quality grades of base liquor were analyzed to investigate the relationship between the quality and the key compounds in SSAB. In this study, samples were evaluated by the sensory and further analyzed by headspace solid-phase microextraction gas chromatography-mass spectrometry (HS-SPME-GC-MS) coupled with electronic nose (E-nose). First, by sensory evaluation, the sauce, floral and fruity, fermented aromas and taste indicators (softness, fullness, harmony, purity and persistence) were positively correlated with the quality grade of the base liquor. The E-nose could distinguish the different quality grades of base liquor well. Second, differential compounds were identified via untargeted metabolome based on the HS-SPME-GC-MS. 16 common differential compounds were shared in the base liquor from different fermentation rounds, including 11 esters, 1 alcohol, 2 aldehydes and 2 ketones. It was found that the higher the quality grade of the base liquor, the richer the content of aromatics, alcohols, aldehydes and ketones. The principal component analysis (PCA) biplots of the differential compounds in the different quality grades of base liquor indicated that the superior-grade base liquor has a strong fruity aroma. By correlation analysis of the differential compounds and sensors responses of E-nose, furfuryl ethyl ether, butanoic acid ethyl ester, isopentyl hexanoate, nonanoic acid ethyl ester and 3-methyl-1-butanol had a significant effect on the response intensity of E-nose sensors. In the present study, the key differential compounds between the different quality grades of base liquor were identified, and the sensory differences between the base liquor were digitized.

## 1. Introduction

**Figure 1:**
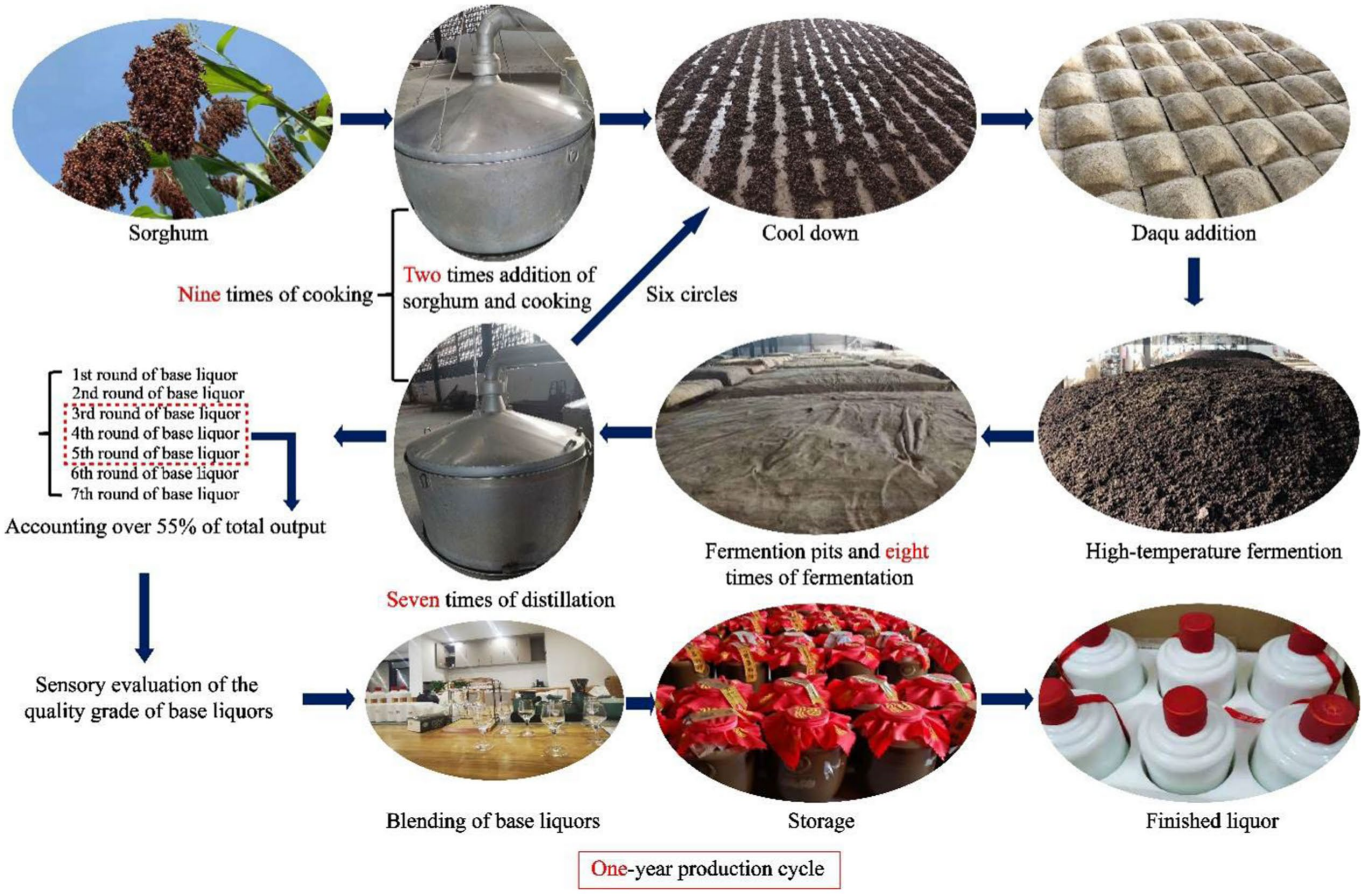
The main processes of soy sauce-aroma type baijiu.

Along with brandy, whisky, gin, vodka and rum, Chinese baijiu is one of the world’s six favorite distilled spirits and is popular with consumers worldwide (1). Soy sauce-aroma type baijiu (SSAB) is one of “the brightest pearls” among the many Chinese baijiu due to its unique flavor and production process (2). The production technology of SSAB is complex and unique. The process flow schematic is shown in Figure 1. It can be briefly summarized as “two-round sorghum addition, the nine-round cooking of sorghum and/or fermented grains (also called “jiupei”), eight-round fermentation and seven-round distillation to obtain the different flavors and quality grades of base liquor, blending and storage to obtain the finished liquor in a one-year production cycle. Compared with other Chinese baijiu processes, high-temperature daqu preparation, high-temperature fermentation, and high-temperature distillation are the most distinctive features of SSAB (3). Sorghum and wheat are the raw materials of baijiu and daqu, respectively.

After half of the total sorghum is cooked and cooled, daqu flour is added in proportion for stacking fermentation. When the stack temperature rises to 48–55°C, the fermented grains are transferred into the pits embedded in the ground and fermented for about 30 days. After that, the raw materials are turned into jiupei to distill the base liquor. The remaining part of sorghum (only the first time) and daqu flour are mixed into the jiupei, and the process of stacking fermentation and cellar fermentation is repeated eight times (4). During repeated fermentations, the seven batches of base liquor with different flavors can be collected from the first to the seventh fermentation round. The base liquor is divided into four grades depending on the quality: superior; first; second and third-grade base liquor, the quality of base liquor is from good to poor quality. The base liquor with different quality grades can become the finished liquor after being combined in proportion. The different quality grades of base liquor from the 3rd, 4th and 5th round of fermentation (RF) have similar sensory attributes, known as “Dahui liquors,” and account for over 55% of the total output in production (5). They can only be identified by a highly skilled taster, which might be subjective and more challenging to produce. What’s more, it is harmful for tasters to taste excessively. In industry, the base liquor obtained through fermentation and distillation is blended and stored to produce the finished liquor. The yield of high-quality base liquor has an important influence on the quality of the finished liquor.

The developed electronic nose (E-nose) can more objectively identify the differences in the quality of liquor and make up for the lack of sensory evaluation. E-nose is a simple and effective detection method for identifying the differences among various samples, which can digitize aromas and effectively distinguish aromas through different sensors (6). The sensory evaluation combined with the E-nose allows for more accurate identification of the quality grades of the base liquor. Hou et al. (7) proposed an E-nose method based on triangular difference-based binary coding to classify the different quality grades of liquor. To investigate the relationship between sensory quality and flavors, and explore the key factors affecting the quality of foods, more and more studies are combining untargeted detection techniques with E-nose, e.g., the analysis of the flavor of Harbin red sausage treated with different processes (8) and the analysis of the flavor of *tamarix* lamb roasted by charcoal and electric (9).

To digitalize the differences in the different quality grades of base liquor, it is essential to analyze the chemical diversity of samples and identify the key compounds. SSAB, like other Chinese baijiu, has many flavors, including organic acids, alcohols, esters, ketones, aldehydes, acetals, lactones, nitrogen and sulfur compounds, and so on (10). Exploring the key flavor components in SSAB has been the focus of many studies. The contribution of various components to the flavor of SSAB was analyzed by the different measurement methods and the appropriate statistical methods. Esters are the main components of flavor compounds in SSAB, most of them display a fruit aroma such as ethyl lactate and ethyl acetate (11). By gas chromatography ion mobility spectrometry (GC-IMS), Cai et al. (12) found ethyl pentanoate, ethyl octanoate, and ethyl butanoate highly contribute to SSAB flavor. Aldehydes can also work with other compounds to form the unique flavor of SSAB, Xiao et al. (13) used gas chromatography–mass spectrometry (GC–MS) to distinguish the different flavor types of Chinese baijiu and found SSAB was positively correlated with furfural and benzaldehyde. Interestingly, the high concentration of pyrazine gives SSAB a special flavor due to its baked, roasted and nutty flavor (14). Niu et al. (15) found that the content of 2, 3, 5-trimethylpyrazine increased during the storage of SSAB. Sulfur compounds are also important to trace compounds in SSAB. Chen et al. (16) used headspace solid-phase microextraction gas chromatography-pulsed flame photometric detection (HS-SPME-GC-PFPD) to investigate the aroma contribution of volatile sulfur compounds in SSAB, 5 sulfur compounds might be the key flavor contributors to SSAB.

Recently, untargeted detection techniques based on GC–MS have become mainstream methods for comprehensively understanding the chemical diversity in liquor (17, 18). The untargeted detection techniques have high sensitivity and a wide detection range (19), and the headspace solid-phase microextraction (HS-SPME) has the advantages of short extraction time and high sensitivity *via* various types of extraction materials (20). Hong et al. (21) applied the untargeted techniques based on HS-SPME-GC-MS to analyze the volatile composition of *soju* and found significant differences in samples. Based on chemical components analysis, different samples can be analyzed with the help of multivariate statistical methods to find critical compounds. Sun et al. (22) used SPME-GC-MS to discriminate the different quality grades of strong-flavor baijiu and found that four esters played a crucial role in grading. Based on GC–MS, Song et al. (23) found that two thiols were important for discriminating the three different flavor types of Chinese baijiu with the help of multivariate statistical methods. Therefore, the untargeted techniques based on HS-SPME-GC-MS were applied in our study.

Identifying the quality of base liquor is a key link in the control of the fermentation process and the quality of finished liquor. Therefore, it is essential to investigate the key compounds of the different quality grades of base liquor and digitalize the quality differences. Most of the previous studies focused on the different components in the different flavor types of finished liquor, but few aimed to investigate the chemical components of the base liquor of SSAB. For example, Xiao et al. (13) selected the finished liquor of SSAB and studied the characterization of different flavor types of Chinese baijiu by GC–MS; Chen et al. (16) investigated the aroma contribution of volatile sulfur compounds in Moutai liquor (finished liquor) by HS-SPME-GC-PFPD. More and more studies are combining HS-SPME-GC–MS with E-nose, which can comprehensively identify the key compounds in foods. Feng et al. (24) identified the differences in volatile compounds of eight kinds of huajiao by using HS-SPME-GC-MS coupled with E-nose. The application of HS-SPME-GC-MS combined with E-nose for identifying the key compounds in SSAB quality grade has not been reported yet.

In the present study, the base liquor from 3rd RF to 5th RF was used to investigate the key aromatics by HS-SPME-GC-MS combined with E-nose. The study was conducted as follows. (1) The samples were classified into different quality grades by sensory evaluation. (2) The results of the sensory evaluation were verified by data-based analysis of the samples by using E-nose. (3) The volatiles of the samples were determined by using an untargeted detection method based on HS-SPME-GC-MS, and (4) the correlation between the sensors of E-nose and the volatiles was revealed the critical differential compounds between the quality grades of base liquor. It is essential for guiding the directional blending and optimizing the storage process to improve the quality of the finished liquor.

## 2. Materials and methods

### 2.1. Chemicals

Anhydrous ethanol (chromatographic grade, ≥99.97%) was purchased from Tianjin kemiou Chemical Reagent Co., Ltd. (Tianjin, China); 2-methyl-2-butanol (≥99.5%) was chromatographic grade standards and purchased from Sinopharm Chemical Reagent Co., Ltd. (Shanghai, China). Alkane standard solutions (C7-C30) were from Sigma-Aldrich (Beijing, China).

### 2.2. Samples

Samples were collected from the most famous region of SSAB production (Renhuai, Guizhou Province, China; 27°33′30″ ~ 28°10′19″N and 105°59′49″ ~ 106°35′50″E). The samples are the base liquor and distilled from the 3rd RF to the 5th RF of jiupei. There were four grades base liquor in each RF, namely superior, first, second, and third-grade base liquor. The ethanol contents of the liquor samples are shown in Supplementary Table S1. Six parallel samples of each grade were collected under the same production conditions. In this way, a total of 72 samples were collected. All samples were stored, sealed and protected from light at room temperature.

### 2.3. Analysis of sensory

Ten panelists with excellent professional qualifications were organized to conduct the sensory evaluation of aromas strictly following the Baijiu flavor wheel of SSAB according to GB/T 33405–2016. The aroma-related indicators and corresponding reference standards include sauce (soybean paste-like aroma), Qu (daqu-like aroma), flower and fruit (fresh flower and fruit-like aroma), distilled grain (steamed sorghum-like aroma), roast (2-ethyl-3, 5-dimethyl pyrazine-like aroma), fermented (ethyl hexanoate-like aroma), grain (sorghum-like aroma), herb (hexanal-like aroma), baked (baked grain cereals-like aroma) and sour (acetic acid-like aroma). The taste indicators include softness, fullness, harmony, purity and persistence. The sensory evaluation was conducted on a five-point scale. The closer the score is to five, the more pronounced the corresponding flavor profile is. The sensory evaluation of each sample was visualized in the radar chart.

### 2.4. Analysis of electronic nose

E-nose analysis was performed with a PEN3 E-nose (Airsense Analytics GmbH, Schwerin, Germany), referring to Zhang et al. (25), with slight modifications. There were ten metal-oxide gas sensors, each of which could reflect different sensory properties depending on its sensitivity to compounds. According to the performance of E-nose, W1C, W5S, W3C, W6S, W5C, W1S, W1W, W2S, W2W, and W3S are sensitive to aromatic compounds; nitrogen oxides; ammonia and aromatic compounds; hydrogen; alkanes and aromatics; methane and a broad range of compounds; sulfur compounds and terpenes; alcohols, aldehydes and ketones; aromatics and organic sulfur compounds; long-chain alkanes, in sequence (9). The 1 ml sample was transferred into a 20 ml headspace vial, capped and allowed to stand at room temperature for 30 min. The equipment must be preheated for more than 30 min before testing and cleared and standardized after each testing. The testing parameters are set as follows: the temperature was 20 ± 2°C; the sample preparation time was 5 s. The measurement time was 45 s, the measurement interval was 1 s, the measurement count was 1 s, the sensor cleaning time was 60 s, and the auto-zero time was 1 s; the intake flow rate was 600 ml/min. Each sample was measured in triplicate.

### 2.5. Analysis of volatile compounds

Following Wang’s (19) method with slight modifications, samples were diluted to 10% (v/v) of ethanol content using redistilled water. 10 mL of diluted liquor, 3.0 g NaCl and 10 μl of 2-methyl-2-butanol (as the internal standard at final content of 8.09 mg/L) were transferred into a 20 mL headspace vial were shocked at 50°C and 280 r/min for 5 min. Then, the volatiles was extracted with an automatic sampler (Supelco, Bellefonte, PA, United States) combined with SPME fiber (DVB/CAR/PDC, divinylbenzene/carboxen/polydimethylsiloxane, 2 cm, 50/30 μm, Supelco Inc., Bellefone, PA, United States) at 50°C for 45 min.

Volatile compounds were analyzed by GC–MS (GC–MS-TQ 8040, Shimadzu Co., Kyoto, Japan) equipped with SH-Rtx-Wax capillary column (30 m × 0.25 mm × 0.25 μm, Shimadzu Co., Kyoto, Japan). The SPME fiber was desorbed for 5 min at an inlet temperature of 250°C. The initial temperature of the column was maintained at 37°C for 5 min, then raised to 45°C at 3°C/min for 5 min, increased to 100°C at 10°C/min for 4 min, warming up to 180°C at 5°C/min for 3 min, and finally raised to 220°C at 10°C/min for 4 min. The injection was set to split mode with a split ratio of 50:1. Helium (purity ≥99.999%) was used as the carrier gas at a 1.0 ml/min flow rate. The ion source adopted an electron ionization source with 70 eV of electron energy. MS transmission line and ion source temperature were 250°C and 230°C, respectively. The mass spectrometry data was collected using full-scan mode (m/z 35–550), and the solvent delay time was 5 min.

After the raw peaks are extracted and processed, the peaks are qualified by matching the mass spectra and retention indexes in NIST17 (United States National Institute of Standards and Technology) database. The retention indexes were calculated by running C7-C30 n-alkanes under the same chromatographic conditions referring to the improved Kovats’s method (26). The identified compounds with match scores above 80 were retained for further analysis. At last, the relative content of each compound was calculated by dividing the isolated compound’s peak area by the internal standard’s peak area (9, 27). Each sample was measured in a sextuplicate.

### 2.6. Statistical analysis of data

Radar charts and principal component analysis (PCA) score plots were drawn by OriginPro 9.0 (MicroCal Inc., MA, United States). Heatmaps were plotted by R 4.2.1 software (Auckland University, Auckland, New Zealand). SIMCA 17.0 (Umetricus Inc., Sweden) calculated the variable importance projection (VIP) values. Compounds with VIP values >1 were identified as differential compounds. Correlation analysis between the sensors’ response values and the differential compounds was calculated according to spearman’s correlation coefficient with R software. When the absolute value of |*ρ*| > 0.6 was considered as a robust correlation (28). The correlative network was visualized with Gephi 0.9.3 (Web Atlas, Paris, France).

## 3. Result and discussion

### 3.1. Sensory evaluation

**Figure 2:**
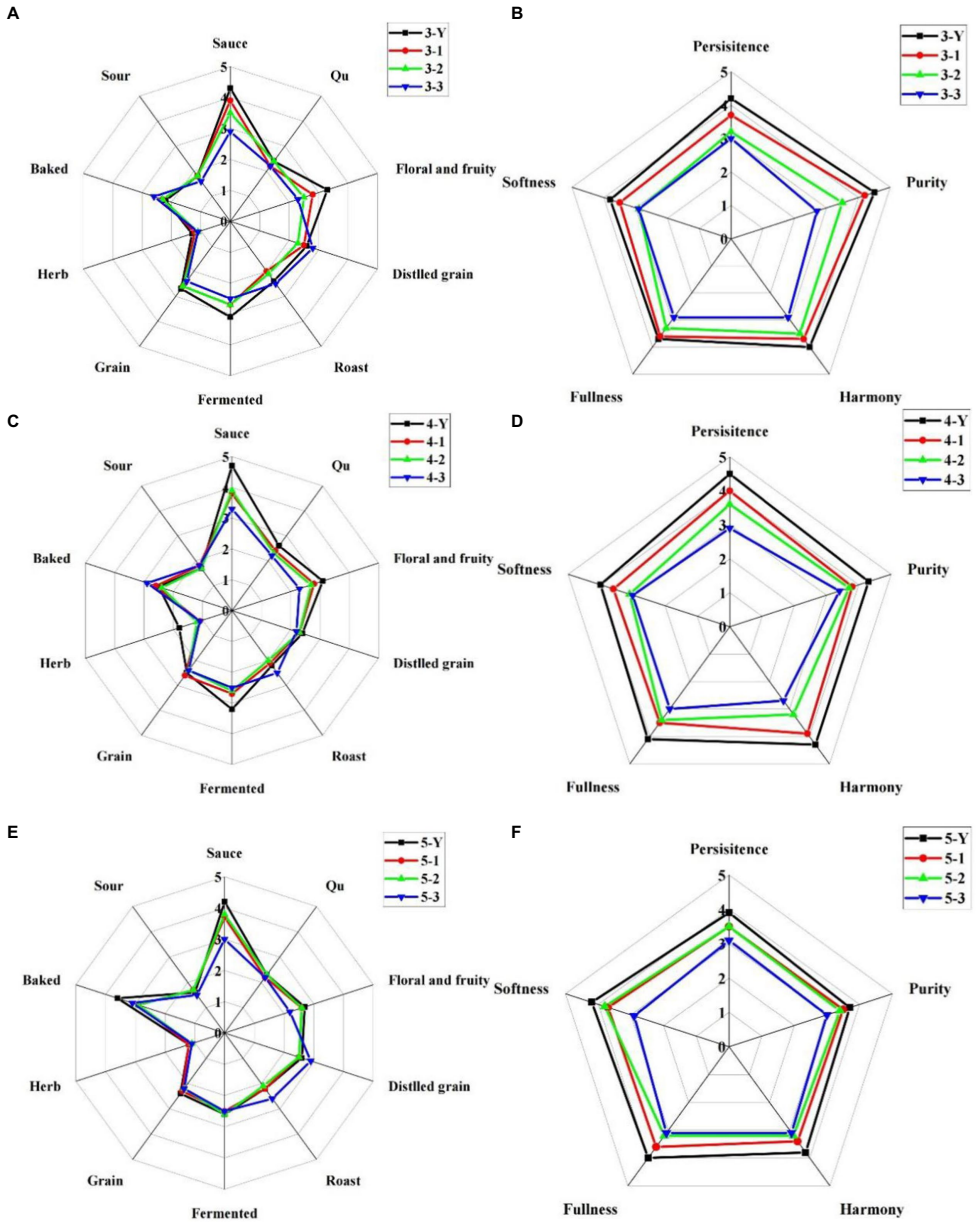
Sensory evaluation of different quality grades of base liquor of three rounds of fermentation, **(A,C,E)** are the aroma profiles of 3rd, 4th, and 5th round of fermentation, respectively; **(B,D,F)** is the taste profiles of 3rd, 4th and 5th round of fermentation, respectively.

The aroma profiles of the different quality grades of base liquor of three RFs are shown in Figures 2A,C,E. The significant aroma-related indicators are sauce, floral and fruity, fermented and baked aromas. Among them, the sauce, floral and fruity, fermented aromas are positively correlated with the quality grade of the base liquor, while the baked aroma is reversed. A baked aroma gives a unique flavor to SSAB, but too much baked aroma will cause an imbalance of flavors. Moreover, the sauce and fermented aromas of 4th RF base liquor are the most outstanding, suggesting the quality of 4th RF base liquor is the best of the three RFs. The taste evaluation of the different quality grades of base liquor of three RFs is shown in Figures 2B,D,F. It showed a trend with the taste indicators in the base liquor of quality grades. The scores of taste indicators are positively correlated with the quality grades of base liquor. The superior-grade base liquor has a full taste with persistence, harmony, softness and purity. On the contrary, the third-grade base liquor has a light taste, a weak aroma and disharmony.

The flavor profiles exhibited by the base liquor from the 3rd RF, 4th RF, and 5th RF are closely related to the process. With the rounds of fermentation and repeated high-temperature cooking of jiupei increased, the intensity of floral and fruity aromas gradually decreased. In contrast, the baked aroma in the liquor became more prominent.

### 3.2. E-nose analysis

**Figure 3:**
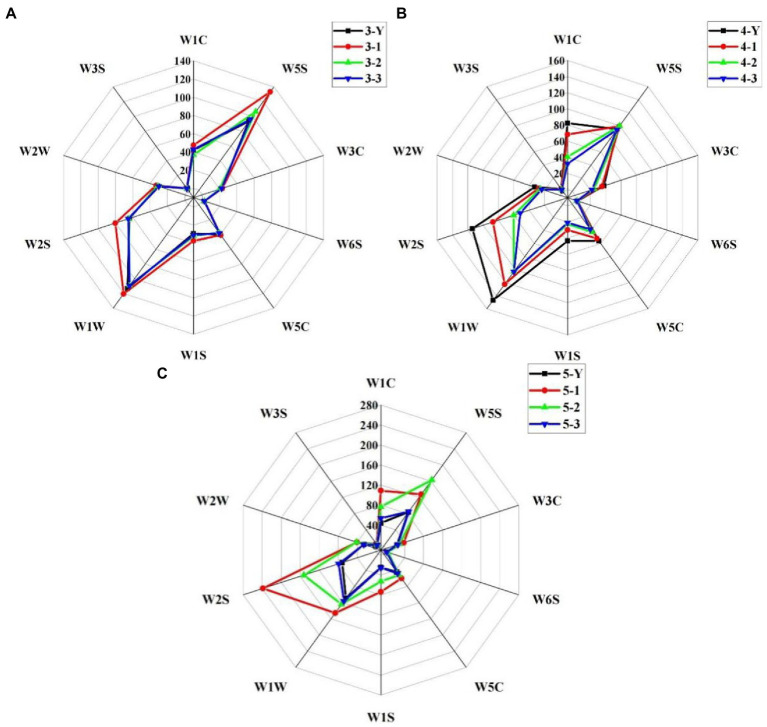
Radar plots of electronic nose (E-nose) sensor response of different quality grades of base liquor of 3rd **(A)**, 4th **(B)**, and 5th **(C)** round of fermentation. W1C, W5S, W3C, W6S, W5C, W1S, W1W, W2S, W2W, and W3S are the number of the E-nose sensor.

The response intensities of sensors are closely related to the content of corresponding compounds in samples. As shown in Figure 3, W1C, W5S, W1W, and W2S sensors have high reaction intensities, which indicates that the samples have high levels of aromatics, nitrogen oxides, sulfur compounds, alcohols, aldehydes and ketones. The response intensities of W1C, W1S, W1W and W2S in the 1st-grade base liquor of 3rd RF and 5th RF is higher than other grade base liquor. Moreover, in the 4th RF base liquor, the response intensity of the W1C, W1S, W1W, and W2S sensors are positively correlated with the quality grade of the base liquor. It is suggested that better quality base liquor has a higher abundance of aromatics, methane, sulfur compounds, alcohols, aldehydes and ketones. Besides, with the number of RF increased, the intensities of W1W and W2S sensor responses gradually increased.

**Figure 4:**
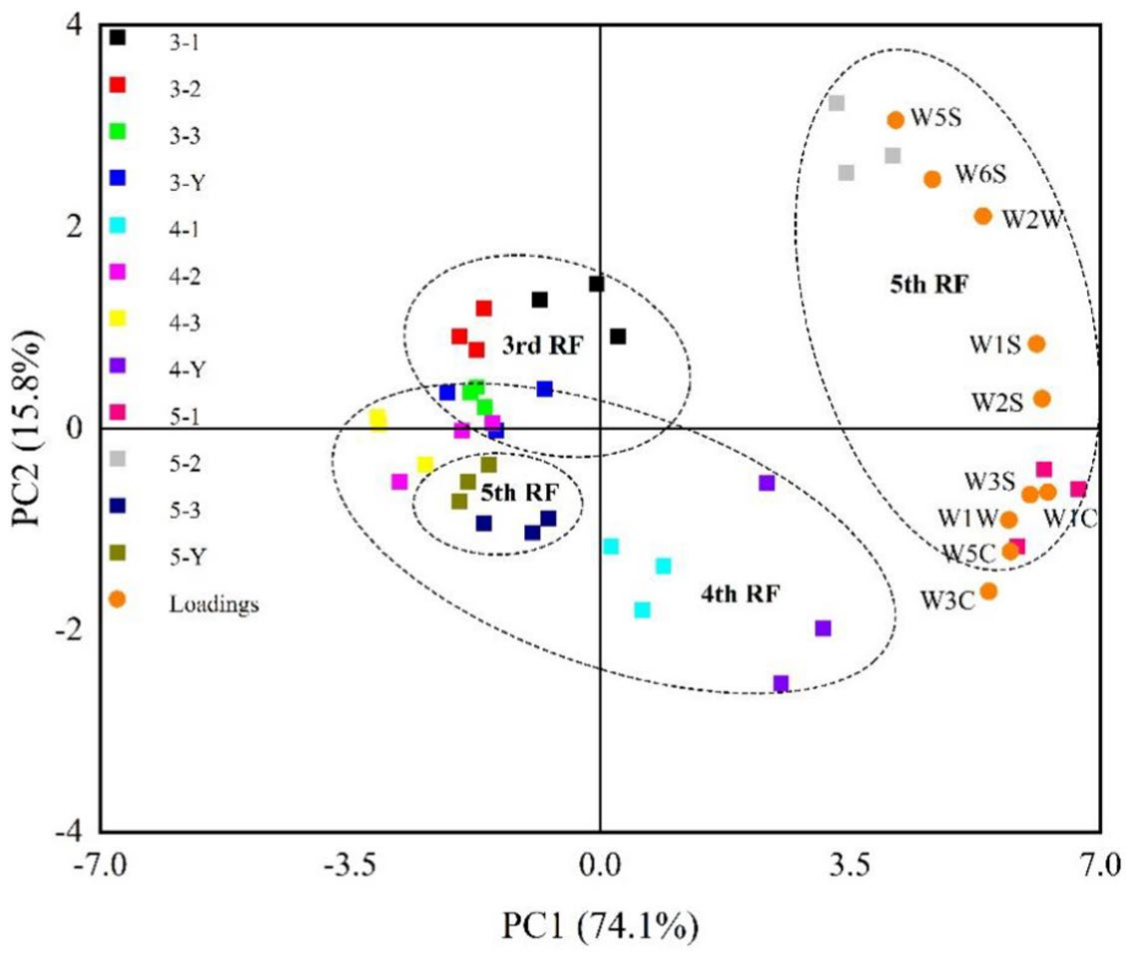
Principal component analysis (PCA) loadings plot of sensors of different quality grades of base liquor.

PCA is a statistical tool used to extract information from variables and explain the differentiation in samples (29). PCA score chart of response intensities is shown in Figure 4. The two principal components can explain 89.9% of the total variance, indicating that E-nose can distinguish the different quality grades of base liquor well. The 3rd and 4th RF samples are close together, while the 5th RF is farther away from the other samples. It indicates that the aroma profile is more similar between the 3rd and 4th RF samples compared to the 5th RF. The 5th RF sample has a distinctive style due to its noticeable baked aroma.

The E-nose is sensitive to the flavors in the sample, and the intensity of sensor response intensity is positively correlated with the relevant volatile compound content in the sample (30). The high-intensity response of most sensors suggests that the composition of SSAB is complex. Sun et al. observed that the unique “sauce” flavor of SSAB was a combination of flavors (31). In similarity to other Chinese baijiu, alcohols and aldehydes are crucial flavors with high content in SSAB (32). In contrast, nitrogen oxides and sulfur compounds are present in small concentrations in SSAB, but they are essential contributors to the distinctive aromas of the liquor. Among the nitrogen oxides, pyrazines are one of the essential Maillard reaction products (33), contributing unique aromas such as baked and nutty aromas (34). Sulfur compounds, a class of compounds with high odor activity at low sensory thresholds, also contribute to SSAB (35). As a result, the W5S sensor exhibits different response intensities in the different grades of the base liquor.

### 3.3. HS-SPME-GC-MS analysis

**Figure 5:**
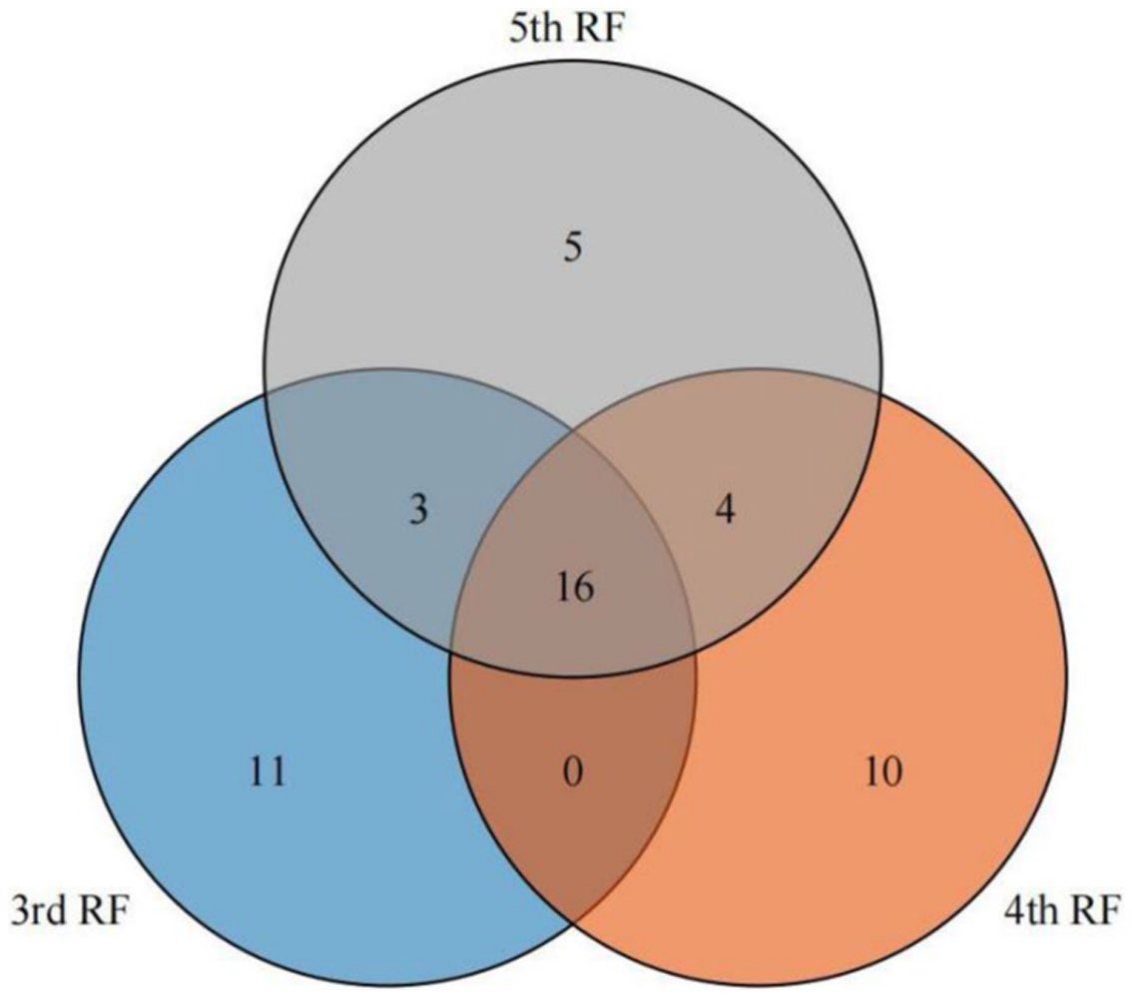
Venn diagram of differential compounds of different quality grades of base liquor of 3rd, 4th and 5th round of fermentation.

**Figure 6:**
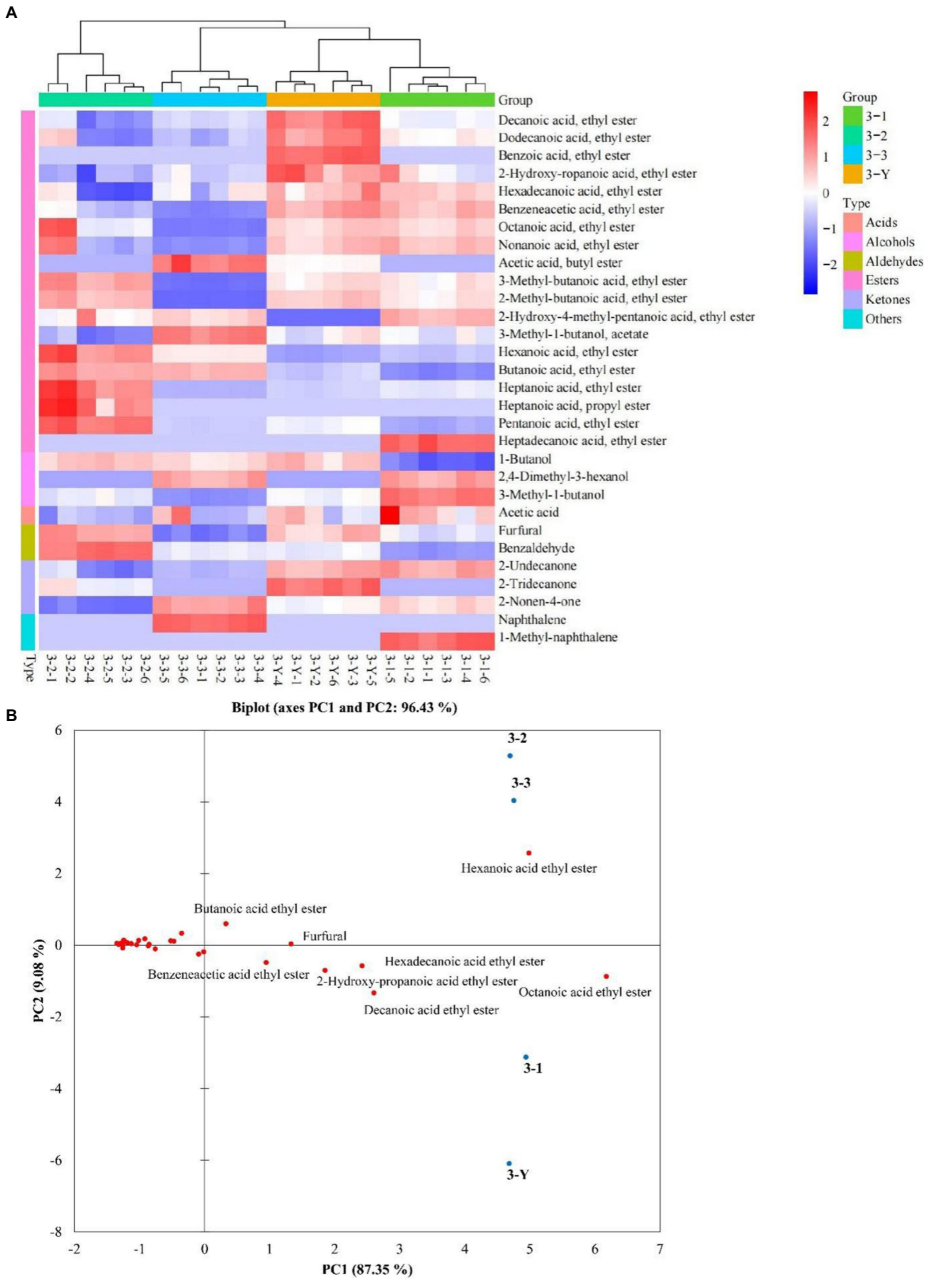
Heatmap **(A)** and principal component analysis (PCA) biplot **(B)** of differential compounds in different quality grades of base liquor of 3rd round of fermentation.

**Figure 7:**
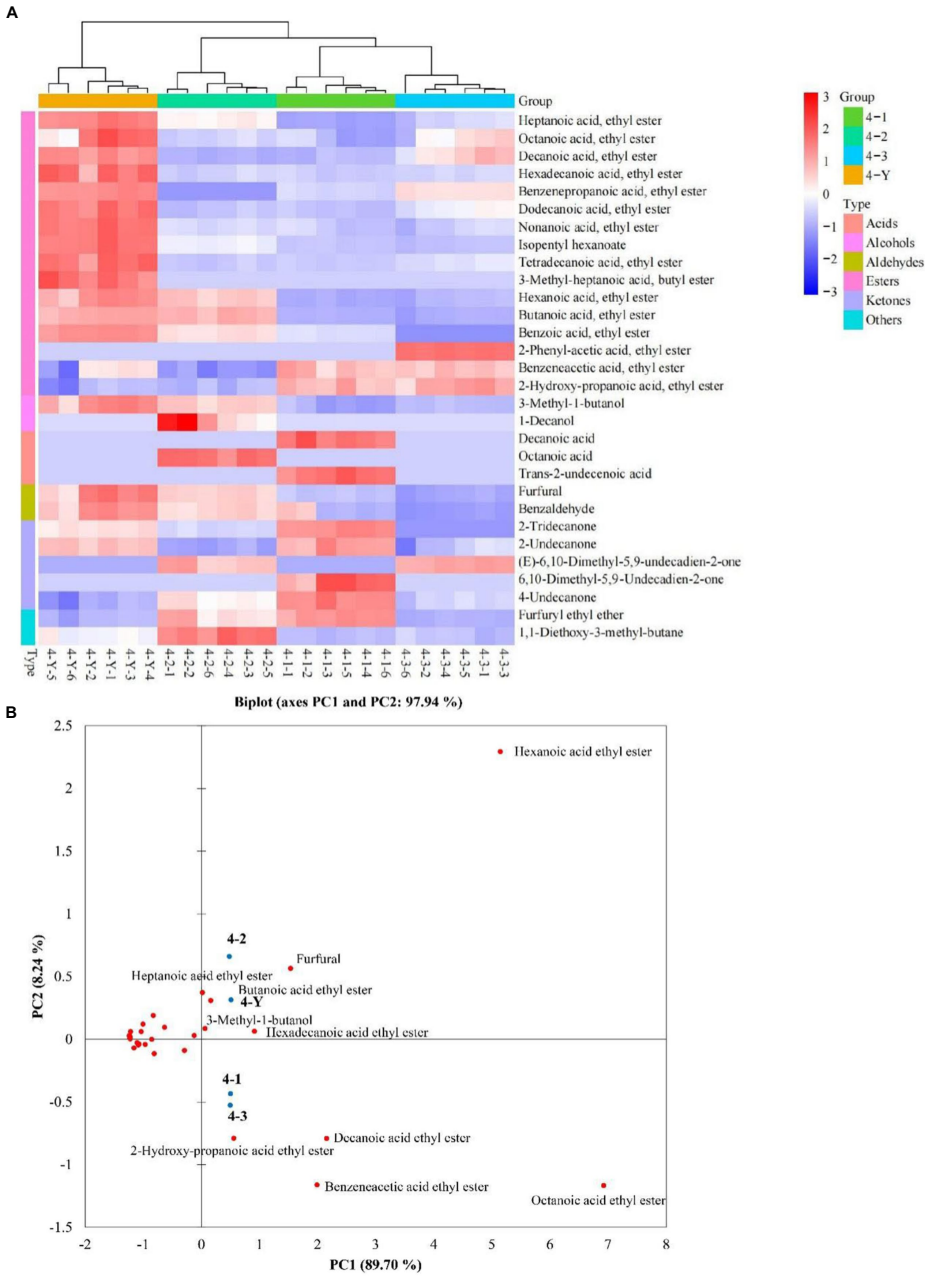
Heatmap **(A)** and principal component analysis (PCA) biplot **(B)** of differential compounds in different quality grades of base liquor of 4th round of fermentation.

**Figure 8:**
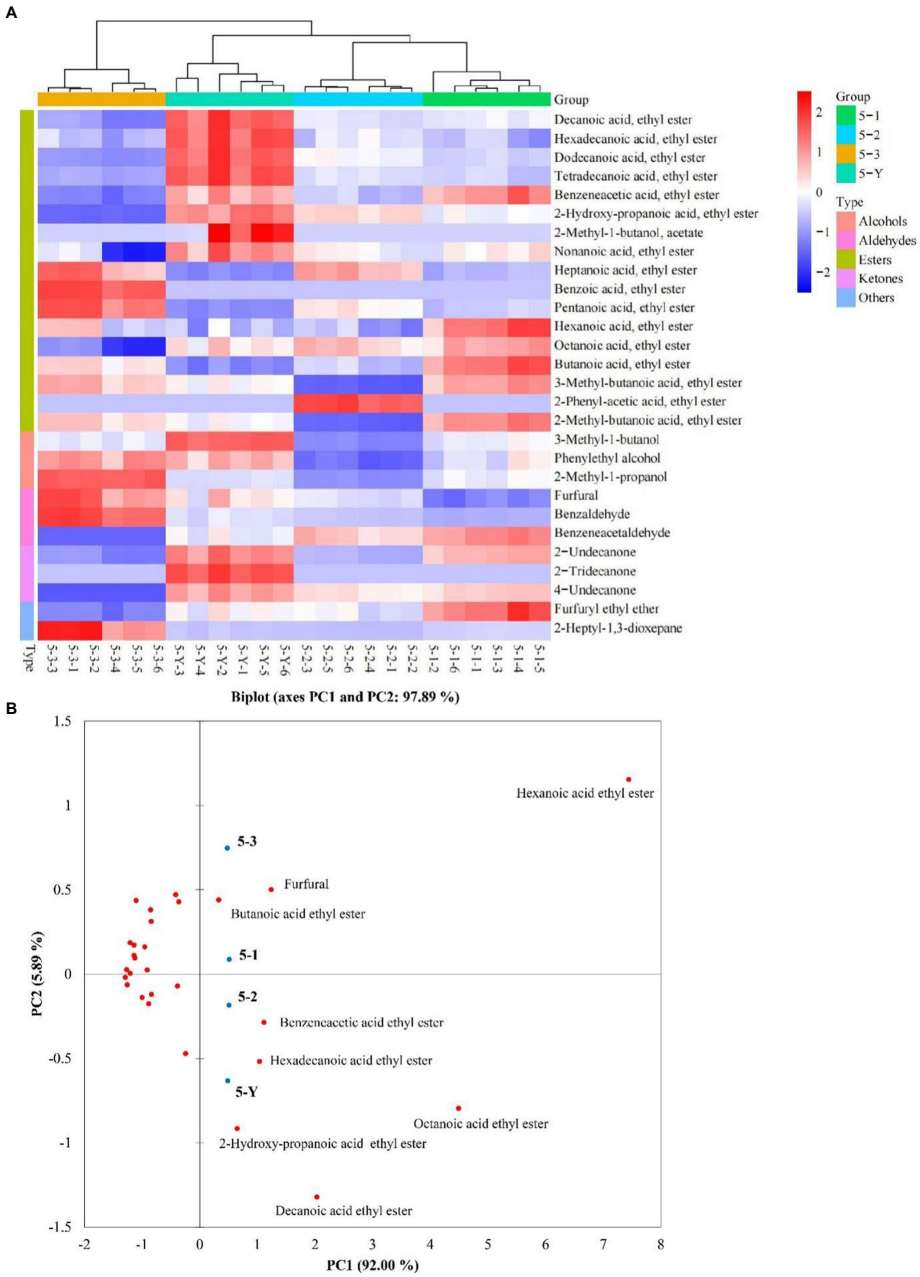
Heatmap **(A)** and principal component analysis (PCA) biplot **(B)** of differential compounds in different quality grades of base liquor of 5th round of fermentation.

A total of 179 compounds were identified in samples by HS-SPME-GC-MS, including 85 esters, 18 alcohols, 7 acids, 11 aldehydes, 16 ketones, 13 alkanes, 4 pyrazines, and 25 others (Supplementary Table S2). VIP values are usually a criterion for screening the differential compounds (28). This study calculated VIP values by the partial least squares-discrimination analysis (PLS-DA) model. The discriminative model was evaluated by a permutation test (*n* = 200). As shown in Supplementary Figures S1A–C, in the three permutation tests, the slope of *R*^2^ in the 3rd, 4th, and 5th RF was 0.0847, 0.304 and 0.0273 (>0), respectively. The intercept of *Q*^2^ on the y-axis in 3rd, 4th, and 5th RF was –0.535, −0.634 and –0.544 (<0.05), respectively. The permutation tests indicated that the PLS-DA model is not overfitting to the data. According to VIP values >1, 30, 30, 28 differential compounds (Supplementary Table S3) in the different quality grades of 3rd RF, 4th RF, and 5th RF base liquor were identified, respectively. As shown in Figure 5 and Table 1, 16 common differential compounds are shared in the base liquor of three RFs, including 11 esters, 2 aldehydes, 1 alcohol, 2 ketones. In order to further observe the performance of differential compounds in the different quality grades of base liquor, Figures 6B, 7B, 8B show the PCA score plots of the differential compounds in the different quality grades of base liquor of 3rd RF, 4th RF and 5th RF, respectively. The PCA biplots explain 96.43, 97.94, and 97.89% of the total variance in the first two principal components, respectively.

**Table 1:** Common differential compounds in different quality grades of base liquor of 3rd, 4th and 5th round of fermentation.

Compounds	CAS	Descriptor aromas	VIP		
			3rd RF	4th RF	5th RF
Butanoic acid ethyl ester	105-54-4	Pineapple (36)	2.389	1.715	2.368
Benzoic acid ethyl ester	93-89-0	Fruity (37)	1.635	1.466	1.802
Benzeneacetic acid ethyl ester	101-97-3	Rosy, honey (38)	1.976	2.232	2.689
Decanoic acid ethyl ester	110-38-3	Fruity, grape (38)	3.988	3.159	3.563
Dodecanoic acid ethyl ester	106-33-2	Sweet, floral (38)	1.461	1.485	2.137
Heptanoic acid ethyl ester	106-30-9	Fruity (38)	2.200	1.618	2.122
Hexanoic acid ethyl ester	123-66-0	Fruity (39)	3.620	3.461	4.307
Hexadecanoic acid ethyl ester	628-97-7	Fruity (37)	2.065	2.629	2.891
Nonanoic acid ethyl ester	123-29-5	Fruity (39)	1.545	1.438	1.067
Octanoic acid ethyl ester	106-32-1	Fruity (38)	3.797	3.849	3.776
2-Hydroxy-propanoic acid ethyl ester	97-64-3	Fruity (39)	2.266	1.923	2.617
3-Methyl-1-butanol	123-51-3	Malty (40)	1.765	1.220	2.301
Benzaldehyde	100-52-7	Almondy (19)	1.315	1.079	1.825
Furfural	98-01-1	Sweet, almondy (39)	1.798	2.483	2.541
2-Tridecanone	593-08-8	Fruity (41)	1.550	1.747	1.338
2-Undecanone	112-12-9	Fruity (41)	1.104	1.539	1.662

#### 3.3.1. Esters

Esters are dominant in the determined compounds. There are 19, 16 and 17 differential esters in the different quality grades of base liquor of 3rd RF, 4th RF and 5 RF, respectively. And there are 11 common differential esters, hexanoic acid ethyl ester, decanoic acid ethyl ester and octanoic acid ethyl ester (VIP > 3) have a significant influence on the quality grade of the base liquor in the three RFs. As shown in Figure 6A, the contents of decanoic acid ethyl ester, 2-hydroxy-propanoic acid ethyl ester, hexadecanoic acid ethyl ester, benzoic acid ethyl ester, benzeneacetic acid ethyl ester and dodecanoic acid ethyl ester in the superior-grade base liquor are higher than the low-grade base liquor in 3rd RF. Figure 6B shows that the superior-grade base liquor in 3rd RF positively correlates with the differential compounds such as decanoic acid ethyl ester (fruity aroma) and 2-hydroxy-propanoic acid ethyl ester (fruity aroma). This trend is consistent with Figure 6A. In Figure 7A, the contents of heptanoic acid ethyl ester, octanoic acid ethyl ester, decanoic acid ethyl ester, hexadecanoic acid ethyl ester, benzenepropanoic acid ethyl ester, dodecanoic acid ethyl ester, nonanoic acid ethyl ester, isopentyl hexanoate, tetradecanoic acid ethyl ester and heptanoic acid 3-methylbutyl ester in the superior-grade base liquor are higher than low-grade base liquor of 4th RF. According to Figure 7B, it can be seen that the superior-grade base liquor in 4th RF is positively correlated with differential compounds such as heptanoic acid ethyl ester (fruity aroma) and hexadecanoic acid ethyl ester (fruity aroma). Figure 8A shows decanoic acid ethyl ester, hexadecanoic acid ethyl ester, dodecanoic acid ethyl ester, tetradecanoic acid ethyl ester, 2-hydroxy-propanoic acid ethyl ester, 2-methyl-1-butanol acetate and nonanoic acid ethyl ester in superior-grade base liquor are higher than low-grades base liquor of 5th RF. According to Figure 8B, we can see that the superior-grade base liquor is positively correlated with the differential compounds such as hexadecanoic acid ethyl ester (fruity aroma) and 2-hydroxy-propanoic acid ethyl ester (fruity aroma). It can be seen that the contents of decanoic acid ethyl ester, hexadecanoic acid ethyl ester and dodecanoic acid ethyl ester are positively correlated with the quality grades of base liquor.

Esters are essential flavors derived from the esterification of carboxylic acids and alcohols, whose contents and compositions contribute to the unique flavor of Chinese baijiu (42). It is not difficult to find that there are a large variety and high concentrations of ethyl esters in liquor; for the high content of ethanol produced during the fermentation of baijiu, ethanol and acids can undergo esterification (38). Moreover, it could be concluded that the superior-grade base liquor has a strongly fruity aroma, which is consistent with the result of sensory evaluation. Many short-chain esters have sweet and fruity aromas (43). Butanoic acid ethyl ester has a pineapple aroma (36), decanoic acid ethyl ester, octanoic acid ethyl ester, hexanoic acid ethyl ester, nonanoic acid ethyl ester and heptanoic acid ethyl ester have a fruity aroma (38, 39). In addition, benzoic acid ethyl ester has a fruity fragrance (37); benzeneacetic acid ethyl ester has a rose and honey fragrance (38). Although most advanced fatty esters have weak aromas (42), they add softness and persistence to the body of liquor and can be considered a volatile marker in different RFs (44). 2-hydroxy-propanoic acid ethyl ester has a fruit aroma but with a high odor threshold value (11), which may be related to the high concentrations in a high-grade base liquor. Song et al. speculated that hexadecanoic acid ethyl ester was a marker of the sauce aroma of SSAB (45). Compared to the low-grade base liquor, the contents of most esters with pleasant odor were higher in the high-grade base liquor. However, other differential esters do not exhibit a clear linear pattern in the different quality grades of base liquor, which might result from the joint contribution of multiple esters in liquor to the flavor in a non-linear relationship. The previous study has found that the high-quality grade liquor contained esters in a suitable ratio (acetic acid ethyl ester:2-hydroxy-propanoic acid ethyl ester: hexanoic acid ethyl ester: butanoic acid, ethyl ester is 4.04:4.19:1.86:1.00) in SSAB (38). However, the unsuitable ratios of esters can lead to spicy and off-flavored liquor. In addition, esters can also work synergistically with alcohols, aldehydes and acids to influence baijiu flavor (11).

#### 3.3.2. Alcohols

Alcohol is mainly produced by yeasts through the glycolytic pathway in the fermentation of grains (46), or converted by aldehydes (47). There are 3, 2 and 3 differential alcohols in the different quality grades of base liquor, 1-butanol, 2, 4-dimethyl-3-hexanol and 3-methyl-1-butanol are the differential alcohols in the different quality grades of base liquor of 3rd RF; decanol and 3-methyl-1-butanol are the differential alcohols in base liquor of 4th RF; phenylethyl alcohol, 3-methyl-1-butanol and 2-methyl-1-propanol are the differential alcohols in base liquor of 5th RF. Among them, 3-methyl-1-butanol is the common differential compound in three RFs, it has a malty aroma and was identified as a critical alcohol marker in SSAB (10, 40). By comparison, it was found that 3-methyl-1-butanol is the least abundant in the 3rd-grade base liquor of 3rd RF, but the highest abundant in the superior-grade base liquor both in 4th RF and 5th RF. We can see that the superior-grade base liquor in 4th RF is positively correlated with 3-methyl-1-butanol in Figure 7B. Meanwhile, phenylethanol is present at higher levels in the superior-grade base liquor than in the lower-grade base liquor of 5th RF, for phenylethanol has a very prominent floral flavor and is an influential contributor to the flavor of various foods (40). It concluded that the contents of 3-methyl-1-butanol and phenylethyl alcohol are positively correlated with the quality grades of base liquor and play a vital role in the classification of the base liquor.

#### 3.3.3. Acids

Acids in baijiu are mainly produced by yeasts and lactic acid bacteria during pit fermentation. The acid contents in SSAB are significantly higher than that of other flavor types of baijiu and have a unique sour flavor (48). Acids were responsible for fruity, cheese and fatty aromas (49). The suitable contents of acids can buffer and harmonize the taste of liquor, while too high contents of acids will generate unpleasant odors (11). In 3rd RF, the acetic acid content is highest in the 1st-grade base liquor. The contents of decanoic acid and trans-2-undecenoic acid are highest in the 1st-grade base liquor, and the content of octanoic acid is highest in the 2nd-grade base liquor of the 4th RF. Among them, decanoic acid has a paint aroma, and octanoic acid has fruit and floral aromas (50). Acetic acid with a high concentration in liquor will be very irritating and bring an unpleasant taste. Interestingly, it can react with ethanol to produce esters (e.g., ethyl acetate) and improve the flavor of liquor (44).

#### 3.3.4. Aldehydes

Aldehydes are also important flavor compounds and have a harmonizing effect on the flavor of baijiu, which comes through the oxidation of alcohol (51). Benzaldehyde and furfural are the common differential aldehydes in the different quality grades of base liquor and have the highest contents in the 2nd-grade base liquor of 3rd RF, the superior-grade base liquor of 4th and the 3rd-grade base liquor of 5th RF. As shown in Figure 7A, the superior-grade base liquor of 4th RF is positively correlated with furfural. However, the 3rd-grade base liquor of 5th RF is also positively correlated with furfural. There is no apparent linear relationship between their content and the quality grade of the base liquor, which might be related to the interconversion between the compounds. Additionally, benzene acetaldehyde has a sweet fruit aroma and is the differential compound of the base liquor of 5th RF.

High-temperature treatment is an essential feature of the production process of SSAB and is the key to distinguishing it from other liquor production. Therefore, with the increase of fermentation batches, the content of Maillard reaction products will gradually increase. Furfural is one of the typical products. Furfural is mainly produced by the thermal decomposition of polypentose in grain hulls, which has the aroma and sweetness of almonds and contributes to the strong caramel flavor in SSAB (39). However, the excessive furfural content can lead to the appearance of burnt flavors. Benzaldehyde and benzene acetaldehyde are very important aldehydes in SSAB. Xiao et al. (13) found that the “sauce” flavor of SSAB was positively correlated with benzaldehyde. Wang et al. (52) found that benzene acetaldehyde might be responsible for the floral aroma of ripe Pu′er tea. Therefore, the contents of furfural, benzaldehyde and benzene acetaldehyde have an important influence on the flavor and the quality grade of the base liquor of SSAB.

#### 3.3.5. Ketones

Ketones are essential flavor substances in food, usually the products of fat and amino acid degradation during fermentation (53). 2-tridecanone and 2-undecanone are the common differential ketones in the base liquor of three RFs, whose contents are higher in the superior-grade base liquor of 3rd RF and 5th RF, in the 1st-grade base liquor of 4th RF. 2-tridecanone and 2-undecanone are responsible for the fruit aroma (41), suggesting that their contents are closely related to the quality grade of the base liquor.

#### 3.3.6. Others

The other differential compounds are naphthalene and 1-methyl-naphthalene; furfuryl ethyl ether and 1,1-diethoxy-3-methyl-butane; furfuryl ethyl ether and 2-heptyl-1,3-dioxepane. Ethyl furfural can be considered an aging flavor compound and is found in the stored base liquor (54). Xiao et al. (49) found that the sauce aroma correlated highly with 1,1-diethoxy-3-methyl-butane. Naphthalene and 1-methyl-naphthalene are benzene derivatives, which can contribute to the phenolic aroma of samples (55). The differential compounds have an important influence on distinguishing the quality grades of base liquor, contributing to the flavor of SSAB.

In conclusion, the composition of SSAB is complex, and only the right content of components will create a harmonious flavor. For instance, octanoic acid has a cheese-like aroma at low concentrations and produces a sour odor at high concentrations (56); the proper content of butanoic acid ethyl ester can make baijiu refreshing and pure, and the excessive content will lead to sweat odor (38).

### 3.4. Correlation analysis of differential compounds and sensors responses of E-nose

**Figure 9:**
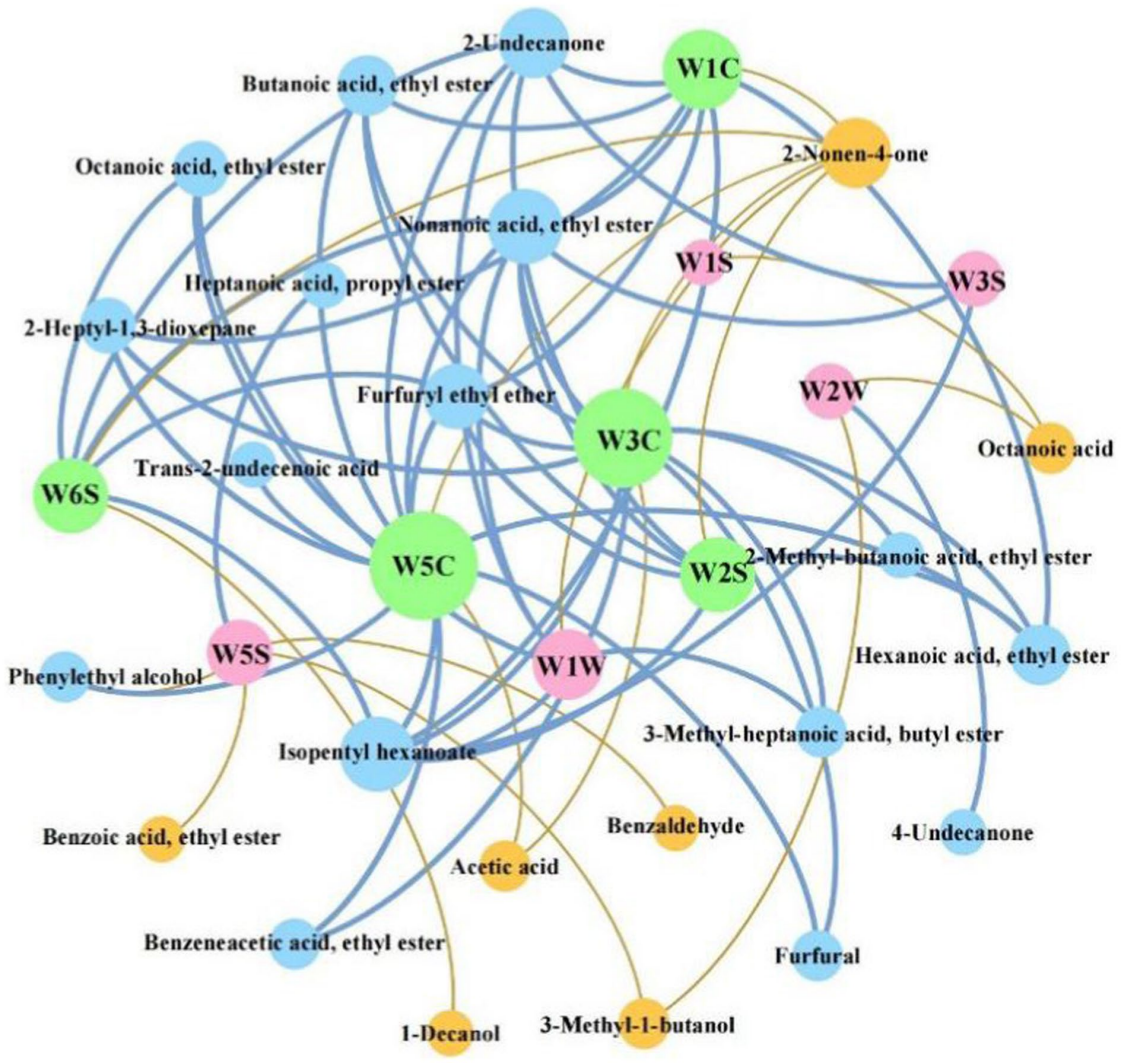
Correlation analysis of differential compounds and sensors responses of electronic nose (E-nose).

Figure 9 established a correlation network of 10 E-nose sensors with the differential compounds. 66 pairs of significant relationships were screened based on |*ρ*| > 0.6, including 50 positive and 16 negative correlations. The compounds closely related to the sensor response intensity are 11 esters, 3 alcohols, 3 acids, 2 aldehydes, 3 ketones, and 2 others. The highest number of compounds is associated with the W5C sensor (14 compounds), followed by the W3S sensor (12 compounds), and are related to the wide range-aromatic property sensors. The compounds of highest correlation with W5C, W3C, W1C, W6S, W2S, W1W, W5S, W2W, W3S, and W1S sensors are furfuryl ethyl ether (*ρ* = 0.879), butanoic acid ethyl ester (*ρ* = 0.882), isopentyl hexanoate (*ρ* = 0.751), nonanoic acid ethyl ester (*ρ* = 0.684), isopentyl hexanoate (*ρ* = 0.753), nonanoic acid ethyl ester (*ρ* = 0.722), 3-methyl-1-butanol (*ρ* = −0.688), octanoic acid (*ρ* = −0.659), 2-undecanone (*ρ* = 0.685), octanoic acid (*ρ* = −0.679), respectively.

The W5C, W3C, W1C, and W2S sensors are all positively correlated with hexanoic acid ethyl ester (fruity aroma), nonanoic acid ethyl ester (fruity aroma), furfuryl ethyl ether (aging aroma) and isopentyl hexanoate (fruity aroma). W5C and W3C sensors are positively correlated with butanoic acid ethyl ester (pineapple aroma), benzeneacetic acid ethyl ester (honey aroma) and furfural (almondy aroma), suggesting that these compounds have a significant impact on the flavor of SSAB. In addition, W5C, W1C, W6S, W1W, W2S, and W3S sensors are positively correlated with 2-undecanone (fruit aroma) and isopentyl hexanoate (apple aroma). However, the W5C and W3C sensors are negatively correlated with acetic acid. It can be seen that most sensors are highly correlated with esters, especially W5C and W3C sensors. These esters were related to the floral and fruity aromas, suggesting that W5C and W3C sensors are susceptible to the floral and fruity aromas.

## 4. Conclusion

During the production of SSAB, the base liquor obtained from the 3rd RF, 4th RF and 5th RF can provide over 55% of the liquor capacity for the finished liquor. Their quality has a significant impact on the quality of the finished liquor. Therefore, it is essential to study the differences between the different quality grades of base liquor and explore the key compounds closely related to the sensory quality for the subsequent base liquor blending and storage process. In the present study, the different quality grades of base liquor from 3rd RF to 5th RF were comprehensively analyzed by HS-SPME-GC–MS combined with E-nose and sensory evaluation. The differences in sensory attributes between the base liquor were analyzed and digitized by sensory evaluation and E-nose. With the help of an untargeted detection technique based on HS-SPME-GC–MS, a comprehensive composition of the samples was detected to identify the key compounds with essential effects on the qualities of SSAB. It was found that 16 differential compounds were shared in the base liquor of the 3rd RF, 4th RF and 5th RF, in which esters were predominant. The principal component analysis (PCA) biplots of the differential compounds in the different quality grades of base liquor indicated that the superior-grade base liquor has a strong fruity aroma. Correlation analysis between the E-nose sensors and the differential compounds found furfuryl ethyl ether, butanoic acid ethyl ester, isopentyl hexanoate, nonanoic acid ethyl ester, 3-methyl-1-butanol, octanoic acid and 2-undecanone were highly correlated with sensors, indicting the key compounds play a crucial role in distinguishing the quality grade of 3rd RF, 4th RF and 5th RF base liquor. Thus, the quantitative data on the sensory difference of the base liquor can be realized through the methods presented in this study. It is significant to identify the key compounds affecting the quality grade of the base liquor to guide the directional blending and the optimization of the storage process for enhancing the quality of finished liquor.

## Data availability statement

The original contributions presented in the study are included in the article/Supplementary material, further inquiries can be directed to the corresponding author.

## Author contributions

JW: methodology, software, formal analysis, investigation, data curation, and writing-original draft preparation. RC: visualization and supervision. XL: methodology and investigation. ZF: software. CX: formal analysis. WZ: formal analysis. CZ: data curation. XW: conceptualization, visualization, writing-review and editing, supervision, project administration, and funding acquisition. All authors have read and agreed to the published version of the manuscript.

## Funding

This research was funded by Guizhou Science and Technology Plan Project (QKHZC-[2022]019).

## Conflict of interest

The authors declare that the research was conducted in the absence of any commercial or financial relationships that could be construed as a potential conflict of interest.

## Publisher’s note

All claims expressed in this article are solely those of the authors and do not necessarily represent those of their affiliated organizations, or those of the publisher, the editors and the reviewers. Any product that may be evaluated in this article, or claim that may be made by its manufacturer, is not guaranteed or endorsed by the publisher.

## References

[1] Zheng XW Han BZ. Baijiu, Chinese liquor: history, classification, and manufacture. J. Ethnic Foods. (2016) 3:19–25. doi: 10.1016/j.jef.2016.03.001

[2] Yang F Liu YF Chen LQ Li JH Wang L Du GC. Genome sequencing and flavor compound biosynthesis pathway analyses of bacillus licheniformis isolated from Chinese Maotai-flavor liquor-brewing microbiome. Food Biotechnol. (2020) 34:193–211. doi: 10.1080/08905436.2020.1789474

[3] Ji KL. The flavor and process characteristics of Moutai-flavor liquors. Food Machin. (1988) 1:12–14+16.

[4] Duan JW Yang SQ Li HH Qin D Shen Y Li HH. Why the key aroma compound of soy sauce aroma type baijiu has not been revealed yet? Lebensm Wiss Technol. (2022) 154:112735:112735. doi: 10.1016/j.lwt.2021.112735

[5] Yang DJ. Styles and flavoring components characteristics and functions of Maotai-flavor liquor of different production turn and production techniques influence. Liquor Making Sci Technol. (2004) 4:112735–7.

[6] Abrodo PA Llorente DD Corujedo SJ de la Fuente ED Alvarez MDG Gomis DB. Characterisation of Asturian cider apples on the basis of their aromatic profile by high-speed gas chromatography and solid-phase microextraction. Food Chem. (2010) 121:1312–8. doi: 10.1016/j.foodchem.2010.01.068

[7] Hou HR Meng QH Qi PF Jing T. A hand-held electronic nose system for rapid identification of Chinese liquors. IEEE Trans Instrum Meas. (2021) 70:1–11. doi: 10.1109/TIM.2021.311278933776080

[8] Yin XY Lv YC Wen RX Wang Y Chen Q Kong BH. Characterization of selected Harbin red sausages on the basis of their flavour profiles using HS-SPME-GC/MS combined with electronic nose and electronic tongue. Meat Sci. (2021) 172:108345. doi: 10.1016/j.meatsci.2020.108345, PMID: 33120175

[9] Xu YJ Zhang DQ Chen RX Yang XY Liu H Wang ZY. Comprehensive evaluation of flavor in charcoal and electric-roasted Tamarix lamb by HS-SPME/GC-MS combined with electronic tongue and electronic nose. Foods. (2021) 10:2676. doi: 10.3390/foods10112676, PMID: 34828957 PMC8623117

[10] Zhu SK Lu X Ji KL Guo KL Li YL Wu CY. Characterization of flavor compounds in Chinese liquor Moutai by comprehensive two-dimensional gas chromatography/time-of-flight mass spectrometry. Anal Chim Acta. (2007) 597:340–8. doi: 10.1016/j.aca.2007.07.007, PMID: 17683748

[11] Wei Y Zou W Shen CH Yang JG. Basic flavor types and component characteristics of Chinese traditional liquors: a review. J Food Sci. (2020) 85:4096–107. doi: 10.1111/1750-3841.15536, PMID: 33190291

[12] Cai WC Wang YR Wang WP Shu N Hou QC Tang FX. Insights into the aroma profile of sauce-flavor baijiu by GC-IMS combined with multivariate statistical analysis. J Anal Methods Chem. (2022) 2022:1–14. doi: 10.1155/2022/4614330 PMC8983223 35392280

[13] Xiao ZB Yu D Niu YW Ma N Zhu JC. Characterization of different aroma-types of Chinese liquors based on their aroma profile by gas chromatography-mass spectrometry and sensory evaluation. Flavour Fragr J. (2016) 31:217–27. doi: 10.1002/ffj.3304

[14] Fan WL Xu Y Zhang YH. Characterization of pyrazines in some Chinese liquors and their approximate concentrations. J Agric Food Chem. (2007) 55:9956–62. doi: 10.1021/jf071357q, PMID: 17970591

[15] Niu YW Chen XM Xiao ZB Ma N Zhu JC. Characterization of aroma-active compounds in three Chinese Moutai liquors by gas chromatography-olfactometry, gas chromatography-mass spectrometry and sensory evaluation. Nat Prod Res. (2017) 31:938–44. doi: 10.1080/14786419.2016.1255892, PMID: 27834102

[16] Chen S Sha S Qian M Xu Y. Characterization of volatile sulfur compounds in Moutai liquors by headspace solid-phase microextraction gas chromatography-pulsed flame photometric detection and odor activity value. J Food Sci. (2017) 82:2816–22. doi: 10.1111/1750-3841.13969, PMID: 29131338

[17] Liu YB Qiao ZN Zhao ZJ Wang X Sun XY Han SN. Comprehensive evaluation of Luzhou-flavor liquor quality based on fuzzy mathematics and principal component analysis. Food Sci Nutr. (2022) 10:1780–8. doi: 10.1002/fsn3.2796, PMID: 35702309 PMC9179129

[18] Choi HU Kim TW Lee SJ. Characterization of Korean distilled liquor, soju, using chemical, HS-SPME-GC-MS, and sensory descriptive analysis. Molecules. (2022) 27:2429. doi: 10.3390/molecules27082429, PMID: 35458627 PMC9028313

[19] Wang LL Fan SS Yan Y Yang L Chen S Xu Y. Characterization of potent odorants causing a pickle-like off-odor in Moutai-aroma type baijiu by comparative aroma extract dilution analysis, quantitative measurements, aroma addition, and omission studies. J Agric Food Chem. (2020) 68:1666–77. doi: 10.1021/acs.jafc.9b07238, PMID: 31957444

[20] Chen JH Tao LN Zhang T Zhang JJ Wu TT Luan DL. Effect of four types of thermal processing methods on the aroma profiles of acidity regulator-treated tilapia muscles using E-nose, HS-SPME-GC-MS, and HS-GC-IMS. Lebensm Wiss Technol. (2021) 147:111585. doi: 10.1016/j.lwt.2021.111585

[21] Hong JM Kim TW Lee SJ. Sensory and volatile profiles of Korean commercially distilled soju using descriptive analysis and HS-SPME-GC-MS. Foods. (2020) 9:1330. doi: 10.3390/foods9091330, PMID: 32967326 PMC7555153

[22] Sun ZB Li JK Wu JF Zou XB Ho CT Liang LM. Rapid qualitative and quantitative analysis of strong aroma base liquor based on SPME-MS combined with chemometrics. Food Sci Hum Wellness. (2021) 10:362–9. doi: 10.1016/j.fshw.2021.02.031

[23] Song XB Zhu L Jing S Li Q Ji J Zheng FP. Insights into the role of 2-Methyl-3-furanthiol and 2-Furfurylthiol as markers for the differentiation of Chinese light, strong, and soy sauce aroma types of baijiu. J Agric Food Chem. (2020) 68:7946–54. doi: 10.1021/acs.jafc.0c04170, PMID: 32615756

[24] Feng XY Wang HW Wang ZR Huang PM Kan JQ. Discrimination and characterization of the volatile organic compounds in eight kinds of huajiao with geographical indication of China using electronic nose, HS-GC-IMS and HS-SPME-GC–MS. Food Chem. (2022) 375:131671:131671. doi: 10.1016/j.foodchem.2021.131671 34865919

[25] Zhang JH Cao J Pei ZS Wei PY Xiang D Cao XY. Volatile flavour components and the mechanisms underlying their production in golden pompano (Trachinotus blochii) fillets subjected to different drying methods: a comparative study using an electronic nose, an electronic tongue and SDE-GC-MS. Food Res Int. (2019) 123:217–25. doi: 10.1016/j.foodres.2019.04.069, PMID: 31284971

[26] Cates VE Meloan CE. Separation of sulfones by gas chromatography. J Chromatogr. (1963) 11:472–8. doi: 10.1016/S0021-9673(01)80948-1 14062606

[27] Zhu M Sun J Zhao HA Wu FH Xue XF Wu LM. Volatile compounds of five types of unifloral honey in Northwest China: correlation with aroma and floral origin based on HS-SPME/GC-MS combined with chemometrics. Food Chem. (2022) 384:132461. doi: 10.1016/j.foodchem.2022.132461, PMID: 35228000

[28] Yang L Fan WL Xu Y. GC × GC-TOF/MS and UPLC-Q-TOF/MS based untargeted metabolomics coupled with physicochemical properties to reveal the characteristics of different type daqus for making soy sauce aroma and flavor type baijiu. Lebensm Wiss Technol. (2021) 146:111416:111416. doi: 10.1016/j.lwt.2021.111416

[29] Dong WJ Hu RS Long YZ Li HH Zhang YJ Zhu KX. Comparative evaluation of the volatile profiles and taste properties of roasted coffee beans as affected by drying method and detected by electronic nose, electronic tongue, and HS-SPME-GC-MS. Food Chem. (2019) 272:723–31. doi: 10.1016/j.foodchem.2018.08.068, PMID: 30309604

[30] Yang WJ Yu J Pei F Mariga AM Ma N Fang Y. Effect of hot air drying on volatile compounds of Flammulina velutipes detected by HS-SPME-GC-MS and electronic nose. Food Chem. (2016) 196:860–6. doi: 10.1016/j.foodchem.2015.09.097, PMID: 26593566

[31] Sun JY Wang Z Sun BG. Low quantity but critical contribution to flavor: review of the current understanding of volatile sulfur-containing compounds in baijiu. J Food Compos Anal. (2021) 103:104079. doi: 10.1016/j.jfca.2021.104079

[32] Li JJ Song CX Hou CJ Huo DQ Shen CH Luo XG. Development of a colorimetric sensor Array for the discrimination of Chinese liquors based on selected volatile markers determined by GC-MS. J Agric Food Chem. (2014) 62:10422–30. doi: 10.1021/jf503345z, PMID: 25289884

[33] Adams A Polizzi V van Boekel M De Kimpe N. Formation of pyrazines and a novel pyrrole in Maillard model systems of 1,3-dihydroxyacetone and 2-oxopropanal. J Agric Food Chem. (2008) 56:2147–53. doi: 10.1021/jf0726785, PMID: 18318495

[34] Hao F Wu Q Xu Y. Precursor supply strategy for Tetramethylpyrazine production by bacillus subtilis on solid-state fermentation of wheat bran. Appl Biochem Biotechnol. (2013) 169:1346–52. doi: 10.1007/s12010-012-0083-0, PMID: 23306895

[35] Yan Y Chen S Nie Y Xu Y. Characterization of volatile sulfur compounds in soy sauce aroma type baijiu and changes during fermentation by GC x GC-TOFMS, organoleptic impact evaluation, and multivariate data analysis. Food Res Int. (2020) 131:109043:109043. doi: 10.1016/j.foodres.2020.109043, PMID: 32247503

[36] Xu YQ Minhazul KAHM Li XT. The occurrence, enzymatic production, and application of ethyl butanoate, an important flavor constituent. Flavour Fragr J. (2020) 35:601–15. doi: 10.1002/ffj.3613

[37] Jia W Fan ZB Du A Li YL Zhang R Shi QY. Recent advances in baijiu analysis by chromatography based technology-a review. Food Chem. (2020) 324:126899:126899. doi: 10.1016/j.foodchem.2020.126899, PMID: 32353653

[38] Xu YQ Zhao JR Liu X Zhang CS Zhao ZG Li XT. Flavor mystery of Chinese traditional fermented baijiu: the great contribution of ester compounds. Food Chem. (2022) 369:130920. doi: 10.1016/j.foodchem.2021.130920, PMID: 34461518

[39] Fan HY Fan WL Xu Y. Characterization of key odorants in Chinese Chixiang aroma-type liquor by gas chromatography-Olfactometry, quantitative measurements, aroma recombination, and omission studies. J Agric Food Chem. (2015) 63:3660–8. doi: 10.1021/jf506238f, PMID: 25797496

[40] Niu YW Yao ZM Xiao Q Xiao ZB Ma N Zhu JC. Characterization of the key aroma compounds in different light aroma type Chinese liquors by GC-olfactometry, GC-FPD, quantitative measurements, and aroma recombination. Food Chem. (2017) 233:204–15. doi: 10.1016/j.foodchem.2017.04.103, PMID: 28530568

[41] Zhang SW Wang T Zhang YM Song B Pang XY Lv JP. Effects of Monascus on proteolysis, lipolysis, and volatile compounds of camembert-type cheese during ripening. Foods. (2022) 11:1662. doi: 10.3390/foods11111662, PMID: 35681411 PMC9180517

[42] Liu HL Sun BG. Effect of fermentation processing on the flavor of baijiu. J Agric Food Chem. (2018) 66:5425–32. doi: 10.1021/acs.jafc.8b00692 29751730

[43] Kohno Y Makino T Kanakubo M. Effect of phase behavior for ionic liquid catalysts with reactants/products on reactivity of esterification from long-chain fatty alcohols and fatty acids. Fluid Phase Equilib. (2019) 490:107–13. doi: 10.1016/j.fluid.2019.03.001

[44] Cai XM Shen Y Chen MY Zhong MY Zhou YL Luo AM. Characterisation of volatile compounds in Maotai flavour liquor during fermentation and distillation. J Inst Brew. (2019) 125:453–63. doi: 10.1002/jib.581

[45] Song Y Xie LY Li JH Shen ZY. Characterization of organic matter in sauce-aroma Chinese liquors by GC-MS and high resolution mass spectrometry. International Conference on Energy, Environment and Bioengineering (ICEEB). Electr Network, (2020) 185:04021, 07-09 AUG 2020

[46] Fan WL Shen HY Xu Y. Quantification of volatile compounds in Chinese soy sauce aroma type liquor by stir bar sorptive extraction and gas chromatography-mass spectrometry. J Sci Food Agric. (2011) 91:1187–98. doi: 10.1002/jsfa.4294, PMID: 21384368

[47] Fan WL Qian MC. Headspace solid phase microextraction and gas chromatography-olfactometry dilution analysis of young and aged Chinese “Yanghe Daqu” liquors. J Agric Food Chem. (2005) 53:7931–8. doi: 10.1021/jf051011k, PMID: 16190652

[48] Song ZW Du H Zhang Y Xu Y. Unraveling Core functional microbiota in traditional solid-state fermentation by high-throughput amplicons and Metatranscriptomics sequencing. Front Microbiol. (2017) 8:1294. doi: 10.3389/fmicb.2017.01294, PMID: 28769888 PMC5509801

[49] Xiao ZB Yu D Niu YW Chen F Song SQ Zhu JC. Characterization of aroma compounds of Chinese famous liquors by gas chromatography-mass spectrometry and flash GC electronic-nose. J Chromatogr. (2014) 945-946:92–100. doi: 10.1016/j.jchromb.2013.11.032 24333641

[50] Fan WL Xu Y. Determination of odor thresholds of volatile aroma compounds in baijiu by a forced-choice ascending concentration series method of limits. Liquor Making. (2011) 38:80–4.

[51] Zhang XJ Meng LJ Lu ZM Chai LJ Wang ST Shi JS. Identification of age-markers based on profiling of baijiu volatiles over a two-year maturation period: case study of Lu-flavor baijiu. Lebensm Wiss Technol. (2021) 141:110913:110913. doi: 10.1016/j.lwt.2021.110913

[52] Wang B Meng Q Xiao L Li RL Peng CH Liao XL. Characterization of aroma compounds of Pu-erh ripen tea using solvent assisted flavor evaporation coupled with gas chromatography-mass spectrometry and gas chromatography-olfactometry. Food Sci Hum Wellness. (2022) 11:618–26. doi: 10.1016/j.fshw.2021.12.018

[53] Stephan A Steinhart H. Quantification and sensory studies of character impact odorants of different soybean lecithins. J Agric Food Chem. (1999) 47:4357–64. doi: 10.1021/jf990105p, PMID: 10552816

[54] Vanderhaegen B Neven H Daenen L Verstrepen KJ Verachtert H Derdelinckx G. Furfuryl ethyl ether: important aging flavor and a new marker for the storage conditions of beer. J Agric Food Chem. (2004) 52:1661–8. doi: 10.1021/jf035412g, PMID: 15030227

[55] Liu JK Zhao W Li SH Zhang AX Zhang YZ Liu SY. Characterization of the key aroma compounds in Proso millet wine using headspace solid-phase microextraction and gas chromatography-mass spectrometry. Molecules. (2018) 23:462. doi: 10.3390/molecules23020462, PMID: 29461466 PMC6017027

[56] Tao YS Zhang L. Intensity prediction of typical aroma characters of cabernet sauvignon wine in Changli County (China). Lebensm Wiss Technol. (2010) 43:1550–6. doi: 10.1016/j.lwt.2010.06.003

