# Supplementary material for: Comprehensive identification of key compounds in different quality grades of soy sauce-aroma type baijiu by HS-SPME-GC-MS coupled with electronic nose
Source: Front Nutr. 2023 Mar 7;10:1132527. doi: 10.3389/fnut.2023.1132527 (PMC10028209; doi:10.3389/fnut.2023.1132527)
Supplement: Supplementary file 1 [file Data_Sheet_1.docx]

Supplementary Material

Comprehensive Identification of Key Compounds in Different Quality Grades of Soy Sauce-Aroma Type Baijiu by HS-SPME-GC-MS Coupled with Electronic nose

Xinying Wu*, Junhai Wu, Renyuan Chen, Xiaobo Li, Zheyang Fu, Chun Xian, Wenwu Zhao, Cheng Zhao

*** Correspondence: Xinying Wu: xywu@gzu.edu.cn**

# Supplementary Figures and Tables

## Supplementary Figures


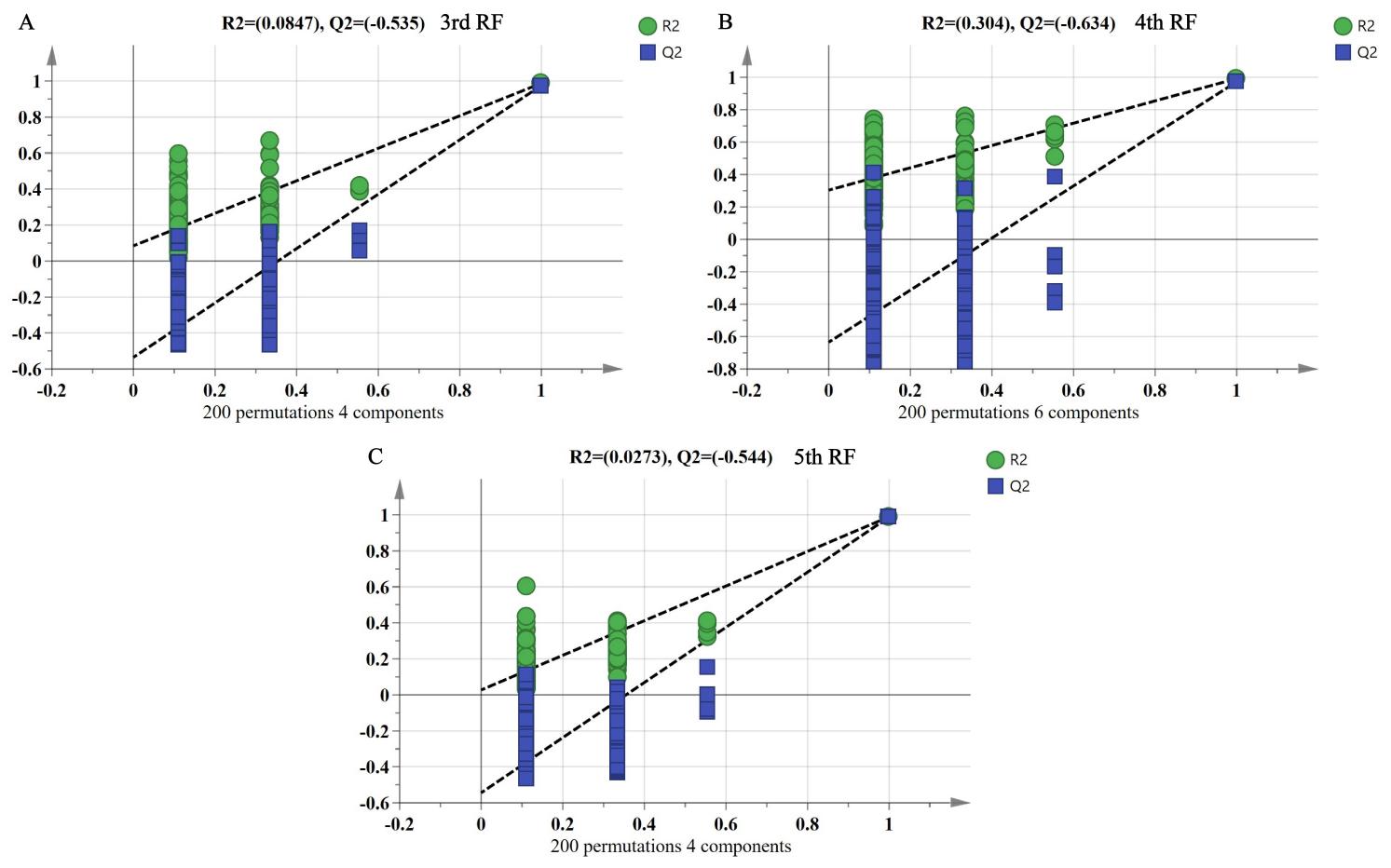


**Supplementary Figure 1.** Results of the permutation test in the different quality grades of base liquor of 3rd (**A**), 4th (**B**) and 5th (**C**) round of fermentation, respectively.

## Supplementary Tables

The supplementary table 1, supplementary table 2 and supplementary table 3 will be uploaded as Excel.
